# Current Treatment Options for Cervical Leiomyomas: A Systematic Review of Literature

**DOI:** 10.3390/medicina57020092

**Published:** 2021-01-21

**Authors:** Federico Ferrari, Sara Forte, Gaetano Valenti, Laura Ardighieri, Fabio Barra, Valentina Esposito, Enrico Sartori, Franco Odicino

**Affiliations:** 1 Department of Obstetrics and Gynecology, Spedali Civili of Brescia, 25123 Brescia, Italy; f.ferrari.obgyn@gmail.com; 2 Department of Clinical and Experimental Sciences, University of Brescia, 25123 Brescia, Italy; sforte88@gmail.com (S.F.); enrico.sartori@unibs.it (E.S.); fodicino@gmail.com (F.O.); 3 Department of General Surgery and Medical-Surgical Specialties, Institute of Obstetrics and Gynecology, University of Catania, 95123 Catania, Italy; valentigaetano@gmail.com; 4 Department of Pathology, Spedali Civili of Brescia, 25123 Brescia, Italy; lauraardighieri@gmail.com; 5 Academic Unit of Obstetrics and Gynaecology, IRCCS Ospedale Policlinico San Martino, 16100 Genova, Italy; 6 Department of Gynecology and Obstetrics, Università degli Studi di Milano, 20122 Milan, Italy; valeespo89@gmail.com

**Keywords:** leiomyoma, cervix, hysterectomy, cervical leiomyoma

## Abstract

*Background and objectives*: Cervical leiomyomas are a rare benign disease. Although they are mainly treated surgically, currently, there is not a standardized treatment for cervical leiomyomas. This study aims to summarize current literature evidence about treatment options for cervical leiomyomas. *Materials and methods*: A systematic research of the literature was conducted in Scopus, PubMed/MEDLINE, ScienceDirect, and the Cochrane Library, including observational prospective and retrospective studies, case series and case reports. We collected data regarding studies related to treatment options for cervical leiomyomas, evaluating the following aspects: study design, population, treatment type, rate of surgical complications, and fertility outcome. *Results*: According to literature research, 38 articles were included. Among 214 patients, the weighted average age was 39.4 years-old; 23 patients were pregnant. Most of the leiomyomas (78%) were extracervical; in 22% of cases (29 patients) were intracervical; 188 patients (88%) received surgical treatment, 6 (3%) received exclusive conservative management and 21 (10%) underwent interventional radiology treatment. One hundred twenty-seven patients (67.5%) underwent myomectomy, while 54 (28.7%) and 7 (3.7%) hysterectomy and trachelectomy, respectively. Cervical myomectomy was performed by open surgery in 21 out of 127 cases (16.5%), while in 92 (72.4%) and 6 (4.7%) patients the surgical approach was performed by traditional and robot-assisted laparoscopy, respectively. The total rate of surgical complications was 5.6%. *Conclusion*: Surgery is the primary therapeutic option for cervical leiomyomas with a low rate of surgical complications. Interventional radiology techniques have reported promising but still limited results.

## 1. Introduction

Uterine leiomyomas (fibroids or myomas) are one of the most common benign smooth muscle tumors in women, having a prevalence of 20–40% after the age of 35 [1,2]. Ninety-five percent of leiomyomas occur in the uterine corpus [3]. Cervical leiomyomas are very uncommon with a frequency of 0.6% [4]; rarely, myomas can be located in other sites of the urogenital tract [5]. Classical symptoms related to myomas are abnormal uterine bleeding (AUB), chronic pelvic pain, dysmenorrhea; bulk-related symptoms can sometimes be observed [6]; similar clinical presentation can also be found in pregnant women affected by uterine leiomyomas [7].

Various classifications of cervical myomas have been proposed in the past years; most of them are based on their location, distinguishing subserosal myomas (defined extracervical type) from myomas located within the cervix (defined intracervical type) [8,9].

The most common therapy for cervical leiomyomas is represented by surgery; however, compared to myomas of the uterine corpus, which are treated with conservative myomectomy [10,11] or hysterectomy [12], the surgical treatment of cervical leiomyomas can be more difficult; this is due to the risk of intraoperative hemorrhage and to the potential injuries to the adjacent organs that are often dislocated and contiguous to the cervical leiomyoma; this may cause a subverted anatomy of the pelvis requiring experienced surgeons [13]; furthermore, the position of the leiomyoma in the cervix poses an extra challenge in the surgical approach in case of a fertility-sparing approach. Currently, a standard surgical treatment for the leiomyomas of the cervix still lacks; therefore, the therapeutic approach depends on the characteristics of the patient, the desire for fertility and the experience of the individual centers and surgeons [14]. In recent years, few experiences of interventional radiology procedures have been reported in those patients interested in preserving their uterus [15].

The aim of this study is to provide an overview of the different treatment options for cervical leiomyomas reported in the literature so far and to give a snapshot of the current state of care for these benign tumors.

## 2. Material and Methods

### 2.1. Study Design

This study is a systematic review of the literature on cervical leiomyomas and their treatments. The review follows the Preferred Reporting Items for Systematic Reviews and Meta-Analyses (PRISMA) guidelines [16].

### 2.2. Inclusion Criteria

The study aimed to ask the following PICOS items.

*Population*: Women with cervical leiomyoma regardless of the co-occurrence of other types of uterine myomas.

*Intervention:* Surgical, radiological, medical, or conservative management for cervical myomas.

*Comparators*: No comparators.

*Outcomes:* To identify the most frequently therapeutic approaches used for treating cervical leiomyomas.

*Study design:* Observational prospective and retrospective studies, case series and case reports were included.

*Language:* Only manuscripts in English language were considered.

### 2.3. Search Strategy

A systematic search of the literature was conducted in Scopus, PubMed/MEDLINE, ScienceDirect and the Cochrane Library from their inception to August 2020. A combination of keywords was used as following “Cervical myoma” OR “Cervical leiomyoma” AND “surgical treatment” OR “alternative treatment” OR “non-surgical treatment” OR “interventional radiology treatment” OR “uterine artery embolization” OR “medical therapy” OR “GnRH agonists” OR “selective estrogen receptor modulator”.

### 2.4. Study Selection and Data Extraction

Two authors (SF and VE) independently screened titles and abstracts from the studies in the search results. The eligible studies were then assessed for inclusion based on their full text. An additional manual search of reference lists was then performed by a third author (GV) not to miss relevant or recent publications. Disagreements on the eligibility of studies were resolved by a fourth author (FF). Three authors (SF, VE and FB) extracted the following data: study features (authors, year of publication, number of cases), population characteristics (age at the diagnosis, fertility status), characteristics of the disease (clinical presentation, radiological features, size and site of myoma, histology in patients underwent surgery), management in case of surgical or conservative treatment (preoperative medical treatment or other types of preoperative procedures, surgical access, intra-operative findings and additional intra-operative procedures, type of treatment), management in case of interventional radiology treatment (type of treatment, volume reduction, efficacy in symptom resolution) and follow-up. Three additional authors (LA, ES and FO) double-checked data extraction. This study was done following the Helsinki Declaration, conforms to the Consensus-based Clinical Case Reporting Guideline Development (http://www.equator-network.org/) the Committee on Publication Ethics (COPE) guidelines (http://publicationethics.org/).

### 2.5. Aim of the Systematic Review

This study aimed to summarize the available evidence in the literature concerning the treatment of cervical myomas.

### 2.6. Data Synthesis

We followed a standardized, pre-specified format for data extraction. The data extracted were evaluated by analyzing the following topics: surgical and conservative treatment for cervical myomas (considering patients’ age at the diagnosis, fertility status, clinical presentation, radiological features, site of myoma, preoperative medical treatment or other types of preoperative procedures, surgical access, intraoperative findings and additional intraoperative procedures, type of treatment, histology, follow-up) and interventional radiology treatment (pregnancy status, age at the diagnosis, symptoms, site, maximum diameter, mean volume and vascularity on aortography of the myoma, type of treatment, myoma mass infarction, volume reduction rate, technique successful and symptoms resolution, follow-up with MRI) for cervical myomas. Depending on their position relative to the endocervical canal, the lesions were classified as extracervical (including subserosal and intramural cervical myomas) and intracervical. Since there was significant heterogeneity between studies, quantitative data synthesis was not possible.

## 3. Results

### 3.1. Systematic Review of the Literature

**Figure 1:**
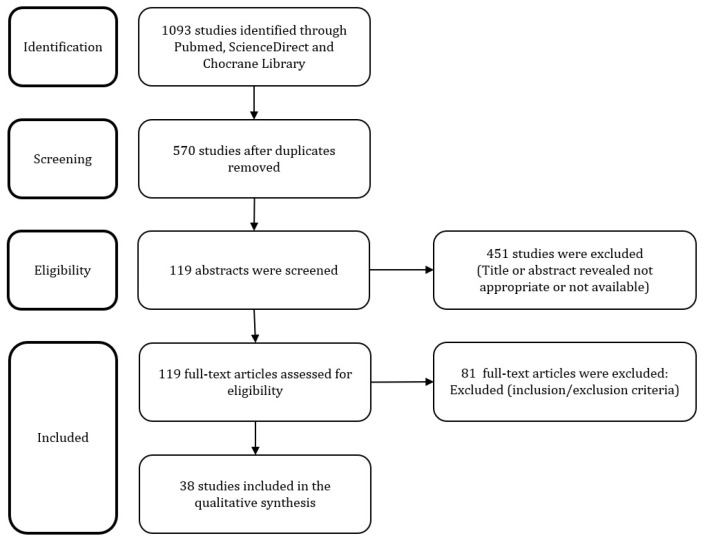
Flowchart of the systematic review.

The search strategy provided a total of 570 articles, after removing duplicates. After screening of manuscript titles, 119 full text and abstracts were considered eligible. Of these, 81 studies were subsequently excluded after the examination of the abstract and full text: 29 manuscripts were excluded because only the abstract was retrieved, 24 as they did not have English language and 28 because they did not have an adequate population and intervention. Definitively, 38 articles were included in our systematic review (Figure 1) [3,5,6,7,8,9,10,11,12,13,14,15,16,17,18,19,20,21,22,23,24,25,26,27,28,29,30,31,32,33,34,35,36,37,38,39,40]. Data synthesis was available in Table 1 and Table 2.

### 3.2. Characteristics of Patients, Symptoms and Radiological Findings

A total of 214 women were included. The average age values were not retrievable in three articles [17,18,19] and were thus only available for 187 patients; the weighted average age was 39.4 years old. Fertility status of the patients was obtained from 24 studies [4,8,19,20,21,22,23,24,25,26,27,36,37,38,39,40] in 57 patients; among them, 28 (49%) patients were in premenopausal status, 5 (9%) in postmenopausal status, 23 patients (40%) were pregnant, and one patient (2%) was in puerperal status.

The description of the symptoms was retrievable in 32 studies [8,17,19,20,21,22,24,25,29,33,34,35,36,37,38,40,41,42,43,44] enrolling 130 patients.

Overall, chronic pelvic or lower back pain was described in 19 patients (14.6%). Fourteen women (11%) suffered from dysmenorrhea, 4 patients (3%) from dyspareunia, and 1 patient (0.7%) from dyschezia; 5 women (4%) complained, instead, of increasing abdominal distension. In 57 patients (44%), cervical leiomyoma caused AUB as hypermenorrhea, intermenstrual or postmenopausal vaginal bleedings; vaginal discharge was reported in 2 patients (1.5%). Chronic urinary complaints were reported in 14 patients (11%) with a percentage of urinary frequency of 93% (13 out of 14 patients). Concerning systemic implications, weight loss was described in 1 patient (0.7%) and anemia in 5 (4%) patients. Twenty-six (20%) women had bulky-related symptoms: particularly, 2 episodes (1.5%) of acute urinary retention, 8 cases (6%) of tenesmus and one (0.7%) case of unilateral hydronephrosis were reported. In 3 cases (2%), an acute spontaneous myoma expulsion was reported and clinically occurred by symptoms like fever, increased volume of a mass protruding from the vagina, pelvic pain, vaginal bleeding, and urinary urgency, frequency, straining, and difficult micturition. Cervical leiomyoma was associated with infertility in 6 patients (4.6%) and pregnancy loss in 3 women (2%). Ten patients (7.7%) were asymptomatic. Data about the clinical impact of cervical myoma on pregnancy and delivery are insufficient, and they were retrievable only for 5 out of 22 pregnant patients (23%); this was because of the absence of information regarding the clinical manifestation of the cervical leiomyoma in the largest study including 17 pregnant patients [45]; for this reason, we described the symptoms of cervical myomas during pregnancy along with those of the other patients; overall, we identified two cases of obstructed labor.

Radiological findings were reported for 183 patients; the diagnosis of cervical leiomyoma was made by ultrasound, magnetic resonance imaging (MRI) and computed tomography (CT) alone in 89 (48.6%), 71 (39%) and 2 (1%) women respectively; in 3 cases (1.6%) the information obtained with ultrasound was integrated with MRI and in 2 other cases (1%) with CT scan. The use of all three of these radiological diagnostic procedures was reported in 15 patients (8%). A further diagnostic study with pelvic angiography was necessary in one case (0.5%) of arteriovenous malformation (AVM) within the cervical leiomyoma. In one out of 89 (1%) cases, the ultrasound scan alone missed the correct diagnosis of cervical leiomyoma [46]. The largest size was reported in 74 patients; in this group, the weighted average of maximum size was 9 cm. We identified 3 cases (1.4%) of leiomyoma of the cervical stump in patients who previously underwent supracervical hysterectomy.

### 3.3. Location of the Cervical Leiomyomas

In 131 patients, lesions were classified as extracervical or intracervical. Overall, 78% (*n* = 102) of the leiomyomas were extracervical and 22% (*n* = 29) were intracervical. In three cases included in the extracervical group (3%) [26,31,33] the mass hanged from the anterior lip of the portio and protruded out from the vagina; the cervical external ostium was completely covered by the myoma, while the cervical canal was not involved and in one of these cases the patient was pregnant at 36 weeks of gestation [26].

In four studies [17,26,44,47] including 63 patients, the location of the cervical leiomyomas was reported neither was deductible from the text, tables or figures.

**Figure 2:**
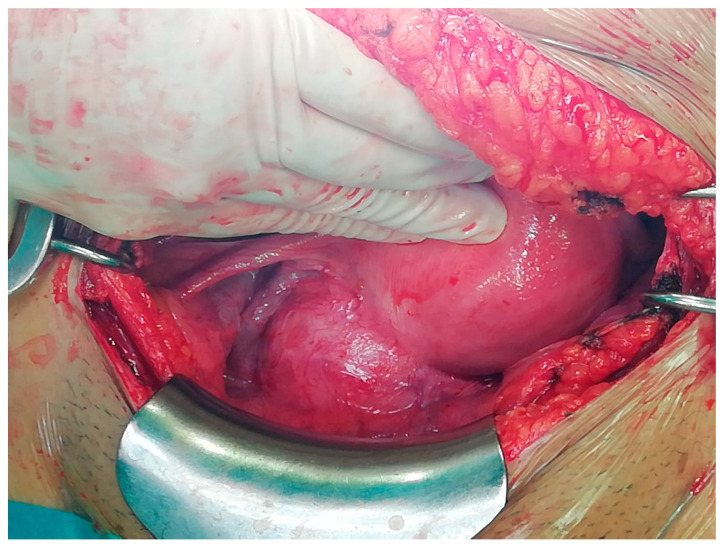
A cervical leiomyoma after surgical laparotomic access that was done by Pfannenstiel-Kerr incision.

Figure 2 described the intraoperative appearance of a cervical leiomyoma during the laparotomic approach done by Pfannenstiel-Kerr incision.

### 3.4. Treatment Options

Overall, 188 patients (88%) underwent surgical treatment, 21 women (10%) interventional radiology treatment and 6 patients (3%) exclusive conservative management. One patient [22] received both surgery and interventional radiology treatments at two different times; therefore, this case was included in both groups. The majority of pregnant women (14 out of 23) were managed by expectant management and underwent surgical treatment after the delivery.

Conservative management after delivery was carried in 26% of cases (6 out of 23); 1 (4.0%) and 2 (8.7%) patients underwent interventional radiology treatment and surgical treatment respectively, during the pregnancy. Forty-three patients were treated by GnRH agonists before the surgery (23%); among them, 5 and 11 women underwent, respectively, laparoscopic myomectomy and laparo-assisted vaginal hysterectomy. No patients received the exclusive use of medical therapy.

#### 3.4.1. Surgical Treatment

Among patients surgically treated, 127 (67.5%) underwent myomectomy, 54 (28.7%) underwent hysterectomy and 7 (3.7%) underwent trachelectomy. Cervical myomectomy was performed by laparotomy in 21 (16.5%) cases; in this group there were 9 surgical procedures (43%) performed at the time of cesarean section after the delivery [48].

Ninety-two (72.4%) and six (4.7%) patients underwent traditional and robot-assisted laparoscopy both for the cervical myomectomy. In one case (0.8%), the laparoscopy access was converted to laparotomy because of the presence of a large submucosal cervical myoma; therefore, there was an overall estimated rate of conversion to laparotomy of 1%. Seven patients (5.5%) underwent vaginal myomectomy for treating four intracervical and three extracervical type leiomyomas; among them, there were two cases of myomectomy in pregnant women at 36 and 15 weeks of gestation [26,30]. The cervical leiomyoma, which was removed in the 36 weeks pregnant woman [26], was an extracervical type; despite the size (55 × 40 millimeters), its removal was possible because there was a broad attachment to the anterior lip of the cervix, which covered the entire cervical opening and protruded in the vagina without involving the cervical canal. An intracervical 50 × 30 × 30 millimeter leiomyoma with a pedicle length of 40 mm originating from the left lateral endocervical canal close to the internal ostium was removed in the other pregnant woman.

In 91 cases (72%), before the myomectomy, diluted vasopressin or epinephrine was injected into the myoma serosa; the surgical access for these patients was laparotomic, laparoscopic and robot-assisted laparoscopic in 10 (11%), 75 (82%) and 6 (7%) cases respectively. Fifty-four women (28.7%) underwent total hysterectomy with or without salpingo-oophorectomy; in 19 patients (35%) a laparotomic hysterectomy, in 13 patients (24%) a laparoscopic-assisted vaginal hysterectomy (LAVH) and in one patient (1.8%) a vaginal hysterectomy was performed. In 21 cases (39%) the surgical access was not reported. In 3 patients (5.5%) the laparotomic hysterectomy was done after delivery at the time of cesarean section. In 85 (45%) cases, the following additional pre or intraoperative procedures for preventing the intraoperative bleedings were employed: bilateral uterine arteries ligation at their origin from the internal iliac artery (*n* = 36) and temporarily blocking of uterine artery blood flow with the use of vessel clips (*n* = 12) during laparoscopic myomectomy; one patient [49] underwent a preventive hypogastric artery ligation before hysterectomy. Bilateral or unilateral internal iliac artery balloon occlusion catheter (IIABOC) was placed before the surgery and eventually inflated and deflated during the surgical procedure in 36 patients (19%); out of them, 25 women underwent hysterectomy (12 by laparotomy and 13 by laparoscopy) and 11 underwent myomectomy (10 by laparotomy and 1 by laparoscopy). Seven patients (3.7%) were treated by trachelectomy with laparotomic or laparoscopic access in 6 (86%) and 1 case (14%), respectively. Three of these patients (43%) had a leiomyoma on the cervical stump; in one of these cases [24], the trachelectomy was performed by laparoscopy.

**Figure 3:**
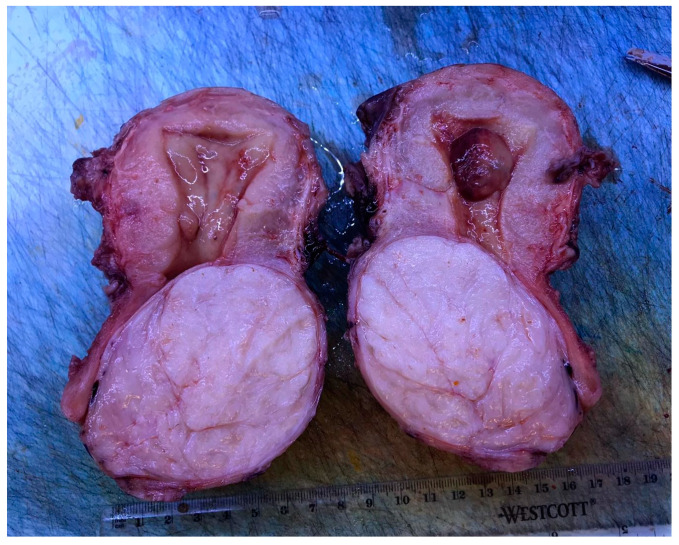
Macroscopic findings of a cervical leiomyoma after hysterectomy.

Histological examination was available in 127 patients and confirmed the diagnosis of leiomyoma in 122 cases (96%). Furthermore, 2 (1.6%) atypical myomas [8,20], 1 (0.8%) AVM in leiomyoma [25], 1 (0.8%) myxoid leiomyoma [26] and 1 (0.8%) case of lipoleiomyoma with focal symplastic features [28] were reported. Figure 3 shows the macroscopic appearance of a cervical leiomyoma.

The complication rate of surgical therapies for cervical leiomyoma was reported in 23 articles: in 125 women, the rate of complications was 5.6% (7 patients experienced complications). Three complications were reported after laparoscopic myomectomy and included one case of paralytic ileus associated with an abscess at the site of the surgical bed (treated with drainage) [41], one case of retroperitoneal hematoma [50], and one case of postoperative fever treated with antibiotic therapy [29]. A patient who underwent laparotomic myomectomy during a cesarean section developed an intraoperative hemorrhage requiring a hysterectomy [45]. Two patients who received LAVH experienced complications: one intraoperative hemorrhage and one postoperative infection treated with antibiotic therapy [29]. Finally, an urinary tract infection was reported in a woman who underwent vaginal hysterectomy [33].

Long-term follow-up was available for 45 patients: there was no recurrence of symptoms in 98% of the women who received surgical treatment. One patient who underwent radical abdominal trachelectomy suffered from pelvic pressure and discomfort for a new cervical leiomyoma being developed by the cervical remnants after one year of follow-up; a subsequent abdominal hysterectomy was carried out [18].

Of note, during the follow-up period, seven patients (15.5%) became pregnant; among them, two and five patients underwent, respectively, vaginal and laparoscopic myomectomy.

#### 3.4.2. Conservative Management

Six patients received exclusive conservative management as they were pregnant [21,45]. One woman underwent an emergency cesarean section for obstructed labor at 37 weeks. Four days after the delivery, after a spontaneous prolapse of the myoma from the vagina, a rubber ring pessary was placed; after 6-week follow-up, the leiomyoma halved its volume not causing significant symptoms [21].

Among the other 5 patients [45], one had a vaginal delivery and 4 were treated conservatively after a cesarean section; two of the latter patients (50%) developed complications; in particular, one woman had persistent fever unresponsive to medical treatment and underwent hysterectomy eight days after the surgery for infection and necrosis of the leiomyoma, endometritis and smooth muscle inflammation; the other patient had a uterine atony provoking an hemorrhage that required an emergency hysterectomy. No data of follow-up were available in this last group of patients.

#### 3.4.3. Interventional Radiology Treatment

Among the 21 women who underwent interventional radiology treatment, one was pregnant at 20 weeks (Table 2) [34]. The largest myoma treated with interventional radiology techniques had a maximum size of 90 mm. One woman received a super-selective uterine fibroid embolization (UFE) and one received a super-selective cervico-vaginal artery embolization. The procedure was successful in 10 out of 18 patients (55.5%) who underwent UAE; a successful outcome was also observed in the patients who underwent UFE and super-selective cervico-vaginal artery embolization.

## 4. Discussion

The surgical treatment of cervical myomas can be challenging and, therefore, require a great experience and expertise of the surgeon; in fact, the presence of a cervical leiomyoma has been identified as an independent factor affecting operation time in minimally invasive surgery [17]. The surgical risks are related to the position of the cervical leiomyoma in the pelvis; in fact, myomas can be very close to the pelvic organs, anteriorly to the bladder, posteriorly to the rectum and bilaterally to the ureters. The leiomyoma can have close relations with these structures, and they can often be strongly adherent and difficult to separate from them, making difficult the identification of a correct cleavage plane for the surgeon; procedures can be further complicated by more restricted and inaccessible surgical spaces [41]. Furthermore, cervical myomas, in particular when large, can alter the position of these structures, subverting the anatomy of the pelvis. Indeed, they can shift the position of the ureter, and engorge the uterine artery and vein, resulting in a high degree of difficulty in performing the surgery [37]. The dislocation of the structures associated with restricted surgical access increases the risk of injuries to the pelvic organs as well as a further difficulty to control major bleeding. Another risk of the surgical treatment of cervical myoma is the intraoperative hemorrhage caused both by the anatomical position that places the cervical myoma adjacent to the arterial and venous uterine vessels and by the neovascularization of the myoma itself [51]. Because it is well known that intraoperative hemorrhage is a significant concern, especially during a myomectomy, various methods were developed to reduce the risk of bleeding [29]; these procedures include the use of preoperative GnRH agonist, tourniquet method, intraoperative injection of vasopressin into the myometrium, and permanent occlusion of the uterine artery [29,51,52,53]. The permanent occlusion of the uterine arteries has been reported to give benefit in reducing hemorrhage during myomectomy as well as achieving a lower rate of disease recurrence; however, it can negatively impact uterine and ovarian function in comparison to the effects of temporary occlusion [29]. Moreover, ligation of the uterine artery can be challenging and sometimes impossible in case of large cervical leiomyomas occupying the entire pelvic cavity and thus causing extremely limited access to the retroperitoneal pelvic space [29]. In our review, bilateral uterine arteries ligation at their origin from the internal iliac artery was performed in 36 patients who underwent laparoscopic myomectomy; a temporary blocking of the uterine artery blood flow with vessel clips was performed in 12 women who also underwent laparoscopic myomectomy; only one patient received a preventive hypogastric artery ligation before the hysterectomy. Furthermore, bilateral or unilateral IIABOC was placed before the surgery in 36 patients: 25 of them underwent hysterectomy and 11 underwent myomectomy (10 on laparotomy and 1 on laparoscopy).

Preoperative medical treatment with GnRH was performed in a low percentage of patients (23%): five of these women underwent laparoscopic myomectomy and 11 underwent LAVH. Conversely, the injection of diluted vasopressin or epinephrine into the serosa before the myomectomy was performed in a greater number of cases (72%).

Globally, the complication rate of surgical procedures for treating cervical myomas was low (5.6%); therefore, the surgical approaches, both conservative and not, should be considered safe when carefully performed by expert hands. Further studies are needed to confirm this evidence. With regards of the patients that got pregnant after either vaginal or laparoscopic myomectomy we were not able to identify favoring factors for the subsequent pregnancy. In fact, nor the size or the anatomical location of the cervical leiomyoma (intra versus extracervical) were factors that can be positively correlated with a successful fertility outcome, given the non-significative distribution in this small subgroup of patients.

Cervical leiomyomas are mostly benign; however, in the presence of a suspected cervical leiomyoma, an atypical or malignant myomatous lesions or solid neoplasms should always be excluded, especially when the lesion presents a large size. In the literature, we have identified a percentage of 1.6% of atypical myomas and 1 case of lipoleiomyoma with focal symplastic features (0.8%). Symplastic leiomyoma is an unusual variant of leiomyoma. It seems that uterine leiomyosarcoma could arise from the preexisting leiomyoma-like areas that often have a symplastic or cellular morphology. As the frequency of leiomyosarcomas is only 0.1–0.3% of all leiomyomas, the progression of myoma to leiomyosarcoma is rarely observed. Because cellular and symplastic leiomyoma-like areas are overrepresented in uterine leiomyosarcoma-associated leiomyoma-like areas, leiomyomas with this morphology may be more candidates to malignant transformation than usual type leiomyomas [28,54]. Thus, it can be said that the symplastic leiomyoma has a low likelihood of malignant transformation and patient counseling is critical to alleviate the anxiety associated with such histologic reports [28].

In addition to surgical therapy, interventional radiology techniques for the treatment of cervical leiomyomas have reported promising but still limited results. In our review, we have found 18 cases of cervical myoma treated with UAE: in particular, one case of super-selective UFE was performed in a pregnant woman and another super-selective cervico-vaginal artery embolization in a 37 years-old woman; in the last case, the patient became pregnant three months after the cervical artery embolization and had a vaginal delivery at 38 weeks without complications [35]. These techniques can be considered in women with a desire to preserve the uterus or who have contraindications to surgery. Although it was showed that pregnancies after uterine embolization have a statistically significantly higher rate for spontaneous abortion (56% vs. 10.5%), risk of malpresentation (20%), and rate of cesarean section (80%) compared to pregnancies after surgical uterine artery occlusion [54], the super-selective embolization of the leiomyoma or cervicovaginal artery is considered a promising option in patients who wish to preserve fertility and/or refuse surgery.

We have not found data on the use of exclusive medical therapy in cervical myomas.

## 5. Conclusions

Hysterectomy or myomectomy according to the patient’s age and childbearing remains the cornerstone in the treatment of cervical leiomyomas. Since surgery can present difficulties, it should be performed by experienced surgeons and can be associated with additional bleeding prevention procedures. Small but promising evidence regards the use of interventional radiology techniques.

## Figures and Tables

**Table 1:** Surgical and conservative treatment of cervical myomas.

Author, Year	Cases	Age at Diagnosis (Years, Mean Age, Range)	Fertility Status	Symptoms	Imaging Assessment	Site of Leiomyoma	Preoperative Procedures	Surgical Access	Type of Treatment	Follow-Up
Hashim, 2020	1	27	Premenopausal	PP; UrS	US; MRI	Extracervical	None	Laparotomy	Myomectomy	Uneventful postoperative course. No recurrence at six months.
Zhang, 2018	1	22	Puerperal	Fever; protruding mass from the vagina	US	Intracervical	None	Vaginal	Myomectomy	Uneventful postoperative course. Pregnancy two years later.
Kaneda, 2017	22	TAH 49.5 (36–62) AM 35.5 (28–40)	Unknown	Unknown	MRI	Extracervical	Placement of bilateral IIABOC	Laparotomy with vasopressin injection	Hysterectomy 12 (54.6%) Myomectomy 10 (45.4%) AM	Uneventful postoperative recovery.
Wong, 2017	1	36	Premenopausal	Bloating abdomen	US; MRI	Extracervical	None	Laparotomy	Trachelectomy	Uneventful postoperative recovery.
Peker, 2016	1	40	Premenopausal	Chronic PP, dyspareunia, primary infertility	US	Extracervical	None	Laparoscopy	Myomectomy	Uneventful postoperative recovery
Giannella, 2016	1	18	Premenopausal	Dyspareunia	US; CT	Extracervical	None	Laparoscopy	Myomectomy	Uneventful postoperative recovery. No recurrence at eight months.
Goel, 2016	1	40	Premenopausal	Bloating abdomen; chronic PP	US	Extracervical	None	Laparotomy	Hysterectomy	Unknown
Peng, 2016	1	42	Premenopausal	UrS	US; CT	Extracervical	None	Laparotomy	Myomectomy	Uneventful postoperative recovery
Garzon-Lopez, 2015	1	31	Premenopausal	ACUR	MRI	Extracervical	None	Laparoscopy with vasopressin injection	Myomectomy	Postoperative abscess at the site of the surgical bed
Bidziński, 2014	1	48	Unknown	ACUR	US	Extracervical	None	Laparotomy	Hysterectomy with preventive hypogastric artery ligation.	Unknown
Gandhi, 2014	1	30	Pregnancy (II trimester)	None	US	Extracervical	None	None	Conservative management Emergency cesarean section for obstructed labor	Spontaneous prolapse out of the vagina four days after surgery. Placement of rubber ring pessary. Halving of volume of the myoma and no symptoms after 6 weeks of follow-up
Keriakos and Maher, 2013	1	29	Pregnancy	Vaginal discharge; AUB; obstructed labor	US	Intracervical	Monolateral uterine artery embolization previous pregnancy.	Vaginal	Conservative management during pregnancy. Emergency cesarean section for obstructed labor and myomectomy six weeks later	Pregnancy at 20 weeks of gestation at follow-up visit.
Kamra, 2013	1	28	Pregnancy (36 weeks)	AUB	US	Extracervical	None	Vaginal	Myomectomy	Unknown
Hsiao, 2013	14	Unknown	Unknown	Unknown	Unknown	Unknown	Unknown	RALM with vasopressin injection in 6 (43%) cases TLM with vasopressin injection in 8 (57%) cases	Myomectomy	Unknown
Ikechebelu, 2012	1	37	Premenopausal	Protruding mass from the vagina. acute PP; AUB; fever; Urs	CT	Intracervical	Broad-spectrum antibiotics	Vaginal	Myomectomy	Uneventful postoperative recovery
Higuchi, 2012	8	35.5 ± 5.3 (29–44)	Unknown	PP; hypermenorrhea; one case of monolateral hydronephrosis	MRI	Extracervical (*n* = 6; 75%) Intracervical (*n* = 2; 33.3%)	GnRH agonists	Laparoscopy with one case of conversion And vasopressin injection	Myomectomy	One case of retroperitoneal hematoma. 50% of childbearing patients got pregnant
Chu, 2012	1	55	Postmenopausal	Intermittent PP; AUB	US	Extracervical	Previous supracervical hysterectomy for leiomyomas	Laparotomy	Trachelectomy	Uneventful postoperative recovery.
Chu, 2012	1	50	Unknown	PP; AUB	MRI	Extracervical	Previous supracervical hysterectomy for leiomyomas	Laparoscopy	Trachelectomy	Uneventful postoperative recovery.
Soeda, 2012	1	55	Postmenopausal	Bloating abdomen	US; MRI; Pelvic angiography showing an AVM in the uterine tumor	Extracervical	Monolateral IIABOC	Laparotomy	Hysterectomy	Unknown
Tian and Hu, 2012	17	32 (28–41)	Pregnancy	Unknown	US	Unknown	Unknown	Laparotomy in 16 (94%) cases	Vaginal delivery 1 (6%) Cesarean section in 16 (94%) cases and 9 of them (56.3%) myomectomy after delivery and 3 (18.8%) hysterectomy after delivery; while 4 (25%) did not receive any treatment	1 (6%) case of intraoperative hemorrhage during myomectomy treated with hysterectomy; 1 (6%) case of persistent fever unresponsive to treatment, underwent hysterectomy eight days later for leiomyoma with signs of infection and necrosis. 1 (6%) case of emergency hysterectomy for postpartum hemorrhage
Pushpalatha, 2011	1	50	Postmenopausal	Lower abdominal heaviness; UrS	CT	Extracervical	None	Laparotomy	Hysterectomy	Uneventful postoperative recovery
Takeda, 2011	13	45.8 (56–36)	Premenopausal (*n* = 11) Postmenopausal (*n* = 2)	Menorrhagia; pressure symptoms	US; MRI; CT	Extracervical (*n* = 9; 69%) Intracervical (*n* = 4; 31%)	GnRH agonist in premenopausal women and placement of bilateral IIABOC	Laparoscopy with bilateral ureteral stenting and epinephrine injection in extracervical cases	LAVH	1 (8%) Intraoperative hemorrhage with blood transfusion. 1 (8%) Post-operative infection
Kilpatrick, 2010	1	32	Unknown	AUB	US	Intracervical	None	Vaginal	Myomectomy at 15 weeks of pregnancy	Uneventful postoperative course. Spontaneous vaginal delivery at 38 weeks gestation and uneventful postpartum course.
Chang, 2010	28	38.0 ± 7.0 (24–52)	Unknown	dysmenorrhea; hypermenorrhoea; UrS; tenesmus; pregnancy loss; infertility	US	Extracervical (*n* = 26; 93%) Intracervical (*n* = 2; 7%)	GnRH agonists in 7% of patients	Laparoscopy with bilateral uterine arteries ligation and vasopressin injection	Myomectomy	Uneventful postoperative recovery. Two infertile patients conceived spontaneously at 1 and 7 months postoperatively
Matsuoka, 2010	16	37.3 ± 4.2 (35–40)	Unknown	hypermenorrhea; pressure symptoms; sterility; lower back pain	MRI	Intracervical (*n* = 5; 31.2%) Extracervical (*n* = 11; 68.7%)	GnRH agonists	Laparoscopy with the injection of vasopressin	Myomectomy with uterine artery clipping in 44% of cases	Uneventful postoperative recovery. One infertile patient got pregnant. No recurrence at four years of follow-up.
Del Priore, 2010	3	Unknown	Unknown	PP	MRI	Extracervical	None	Laparotomy	Radical abdominal trachelectomy	33% recurrent complaints for a new myoma after 12 months
Baum, 2009	1	32	Premenopausal status	PP; protruding mass from the vagina	US	Extracervical	None	Vaginal	Myomectomy	Uneventful postoperative recovery
Sinha, 2009	24	37	Unknown	Menorrhagia (71%)	US	Unknown	Unknown	Laparoscopy with bilateral uterine arteries ligation at their origin in 50% of cases	Myomectomy	Unknown
Takeda, 2009	1	33	Premenopausal	Bloating abdomen; UrS	US; MRI; CT	Extracervical	GnRH agonists; bilateral IIABOC	Laparoscopy with bilateral ureteral stenting	Myomectomy	Post-operative fever No recurrence at 6 years
Takeuchi, 2006	5	36.2 ± 5.3	Premenopausal	Hypermenorrhea; anemia	MRI	Extracervical (*n* = 3; 60%) Intracervical (*n* = 2; 40%)	GnRH agonists	Laparoscopy with temporary clipping of the uterine artery and vasopressin injection	Myomectomy	Uneventful postoperative recovery No recurrence at 2 years
Sengupta, 2006	1	29	Pregnant (36 weeks)	Chronic vaginal discharge; spontaneous expulsion: acute severe PP and AUB	US	Intracervical	None	Vaginal	Myomectomy (after cesarean section)	Uneventful postoperative recovery No recurrence at 6 months
Gurung, 2003	1	37	Premenopausal	Mass protruding from the vagina; AUB	Unknown	Extracervical	None	Vaginal	Hysterectomy	Postoperative UTI
Bajo, 1998	21	50 (47–60)	Unknown	Unknown	US	Intracervical	Unknown	Unknown	Hysterectomy	Unknown
Galt, 1957	1	47	Unknown	Lower back pain, dyschezia, dyspareunia, loss of weight.	Unknown	Extracervical	Previous supracervical hysterectomy	Laparotomy	Trachelectomy	Uneventful postoperative recovery No recurrence at 6 months

PP: pelvic pain; UrS: urinary symptoms; US: ultrasound scan; ACUR: acute urinary retention; AUB: abnormal uterine bleeding; MRI: magnetic resonance imaging; CT: Computer tomography; AVM: Arteriovenous malformation; TAH: total abdominal hysterectomies; AM: abdominal myomectomies; IIABOC: Internal Iliac Artery Balloon Occlusion Catheter; GnRH: gonadotropin-releasing hormone; RALM: robot-assisted laparoscopic myomectomy; TLM: traditional laparoscopic myomectomy; LAVH: Laparoscopic-assisted vaginal hysterectomy; UTI: urinary tract infection.

**Table 2:** Interventional radiology treatment.

Author, year	Cases	Median Age	Fertility Status	Symptoms	Site of Leiomyoma	Imaging Assessment and Size of CL in cm	Mean Volume of CL in mL	Type of Treatment	Myoma Mass Infarction	Volume Reduction Rate (%)	Treatment Success	Symptoms Resolution	Follow Up with MRI
de Bruijn, 2019	8	37	Unknown	AUB; pressure symptoms; PP	Unknown	MRI	Unknown	UAE	90% of patients with solitary CL 60% of patients with concurrent non-cervical myomas	85%	100%	75%	3 months
Kim, 2012	10	Unknown	Unknown	Unknown	Intracervical 8 (80%) Extracervical 2 (20%)	MRI; 6 ± 1.6 cm	92.6 ± 54.8	UAE	20% of cases complete; 60% of cases partial necrosis; 20% of cases no necrosis;	42.8%	20%	Unknown	3 months
Lohle, 2015	1	40	Pregnancy (20 weeks)	AUB	Extracervical	US; MRI	Unknown	UFE	almost complete	Unknown	100%	100%	2 months
DeMeritt, 2019	1	37	Premenopausal	AUB	Extracervical	MRI 3.6 × 3.2 × 4.4 cm	26.54	Super-selective cervico-vaginal artery embolization	100%	91%	100%	100%	Unknown

CL: cervical leiomyoma; AUB: abnormal uterine bleeding; P: intermittent pain not related to the menstrual cycle; UAE: Uterine artery embolization; UFE: Super-selective uterine fibroid embolization.

## Data Availability

Data sharing not applicable.
